# The association between empirical antibiotic regimens and the outcome of peritoneal dialysis-related peritonitis: a multi-center, large-scale cohort study

**DOI:** 10.1007/s40620-025-02437-9

**Published:** 2025-10-24

**Authors:** Yumeng Qiao, Shuang Gao, Shaomei Li, Huaying Pei, Zhaoxia Zheng, Liping Duan, Jinghong Zhao, Ying Zhang, Zibo Xiong, Yumei Liao, Fuyun Sun, Xiaoying Ma, Gang Fu, Shanshan Guo, Tao Zhang, Ying Li, Xianchao Zhang, Xuejian Wang, Caili Wang, Lirong Deng, Zhanzheng Zhao, Jing Xiao, Li Hao, Guiling Liu, Xuanyi Du, Tianrong Ji, Li Zuo, Huiping Zhao, Wenbo Hu, Yulin Li, Yulan Shen, Yong Zhang, Yingli Yue, Shanshan Chen, Lihua Wang, Yan Yan, Beiru Zhang, Rui Yu, Yirong Liu, Xinying Gao, Zhigang Ma, Yingping Li, Xiaoli Chen, Hongyi Li, Shutong Du, Cui Zhao, Zhonggao Xu, Li Zhang, Hongyu Chen, Li Li, Yingchun Ma, Yuanyuan Wei, Jingwei Zhou, Yan Li, Jinwei Wang, Jie Dong, the PDTAP working group

**Affiliations:** 1 Renal Division, Department of Medicine, Peking University First Hospital, Institute of Nephrology, Peking University, Key Laboratory of Renal Disease, Ministry of Health, Ministry of Education, Beijing, China; 2 Renal Division, Department of Medicine, Second Hospital of Hebei Medical University, Hebei, China; 3 Renal Division, Department of Medicine, Handan Central Hospital, Hebei, China; 4 Department of Nephrology, Key Laboratory for the Prevention and Treatment of Chronic Kidney Disease of Chongqing, Chongqing Clinical Research Center of Kidney and Urology Diseases, Xinqiao Hospital, Army Medical University (Third Military Medical University), Chongqing, China; 5 Renal Division, Department of Medicine, Peking University Shenzhen Hospital, Guangdong, China; 6 Renal Division, Department of Medicine, Cangzhou Central Hospital, Hebei, China; 7 Renal Division, Department of Medicine, Peking Haidian Hospital, Beijing, China; 8 Renal Division, Department of Medicine, Third Hospital of Hebei Medical University, Hebei, China; 9 Renal Division, Department of Medicine, Pingdingshan First People’s Hospital, Henan, China; 10 Renal Division, Department of Medicine, First Affiliated Hospital of BaoTou Medical College, Neimenggu, China; 11 Renal Division, Department of Medicine, First Affiliated Hospital of Zhengzhou University, Henan, China; 12 Renal Division, Department of Medicine, Second Affiliated Hospital of Anhui Medical University, Anhui, China; 13 Renal Division, Department of Medicine, Second Affiliated Hospital of Harbin Medical University, Heilongjiang, China; 14 Renal Division, Department of Medicine, Peking University People’s Hospital, Beijing, China; 15 Renal Division, Department of Medicine, People’s Hospital of Qinghai Province, Qinghai, China; 16 Renal Division, Department of Medicine, Beijing Miyun District Hospital, Beijing, China; 17 Renal Division, Department of Medicine, People’s Hospital of Langfang, Hebei, China; 18 Renal Division, Department of Medicine, Second Hospital of Shanxi Medical University, Shanxi, China; 19 Department of Nephrology, Shengjing Hospital of China Medical University, Shenyang, Liaoning, China; 20 Renal Division, Department of Medicine, First People’s Hospital of Xining, Qinghai, China; 21 Renal Division, Department of Medicine, People’s Hospital of Gansu, Gansu, China; 22 Renal Division, Department of Medicine, Taiyuan Central Hospital, Shanxi, China; 23 Renal Division, Department of Medicine, Cangzhou People’s Hospital, Hebei, China; 24 Renal Division, Department of Medicine, First Hospital of Jilin University, Jilin, China; 25 Renal Division, Department of Medicine, People’s Hospital of Chuxiong Yi Autonomous Prefecture, Yunnan, China; 26 Renal Division, Department of Medicine, China Rehabilitation Research Center, Beijing Boai Hospital, Beijing, China; 27 Renal Division, Department of Medicine, Beijing Dongzhimen Hospital, Beijing, China

**Keywords:** Peritoneal dialysis, Peritonitis, Antibiotic regimen, Treatment, Outcome

## Abstract

**Background:**

Peritoneal dialysis (PD)-related peritonitis is a common complication with high morbidity and mortality, and empirical antibiotic regimens vary across countries. Despite some research, inconsistent results and design limitations highlight the need to reassess the association between these regimens and outcomes.

**Methods:**

This study was affiliated with the PD Telemedicine-assisted Platform (PDTAP) study. The primary outcome was peritonitis-associated death, and the secondary outcomes were peritonitis-associated hemodialysis transfer and subsequent peritonitis within 6 months. Propensity score matching and logistic regression were used to access the relationship between empirical antibiotic administration and outcomes.

**Results:**

Altogether, 1431 patients experienced a first episode of peritonitis from June 1, 2016, to April 30, 2019. Among them, 1203 patients were assigned to the cefazolin-based group (*n* = 637) or to the vancomycin-based group (*n* = 566) based on administration of empirical antibiotics against Gram-positive bacteria. Compared to the cefazolin-based group, patients in the vancomycin-based group were older, had a longer PD duration, and reported higher income, along with a greater prevalence of diabetes, cardiovascular disease, and peritonitis history (*P* < 0.05 for all). Both groups exhibited similar rates of peritonitis-associated death and subsequent peritonitis within 6 months (*P* > 0.05 for all), however, the vancomycin-based group was more prone to to hemodialysis transfer (11.00% vs 16.31%, *P* = 0.010, for total peritonitis; 2.62% vs 9.01%, *P* = 0.004, for Gram-positive bacterial peritonitis). After propensity score matching analyses, the rates of death, hemodialysis transfer and subsequent peritonitis remained similar between the two groups (*P* > 0.05 for all). Additionally, multivariate logistic regression, after adjusting for confounders, revealed no significant difference in the outcomes between the two groups (*P* > 0.05 for all). In the sensitivity analysis, excluding culture-negative patients, the results remained similar. In cases of Gram-negative bacterial peritonitis, the use of third-generation cephalosporins also had no association with better prognosis, irrespective of propensity score matching analysis (*P* > 0.05 for all).

**Conclusion:**

Our study suggests that there is no significant difference in prognosis among different empirical antibiotic regimens for peritonitis.

**Graphical Abstract:**

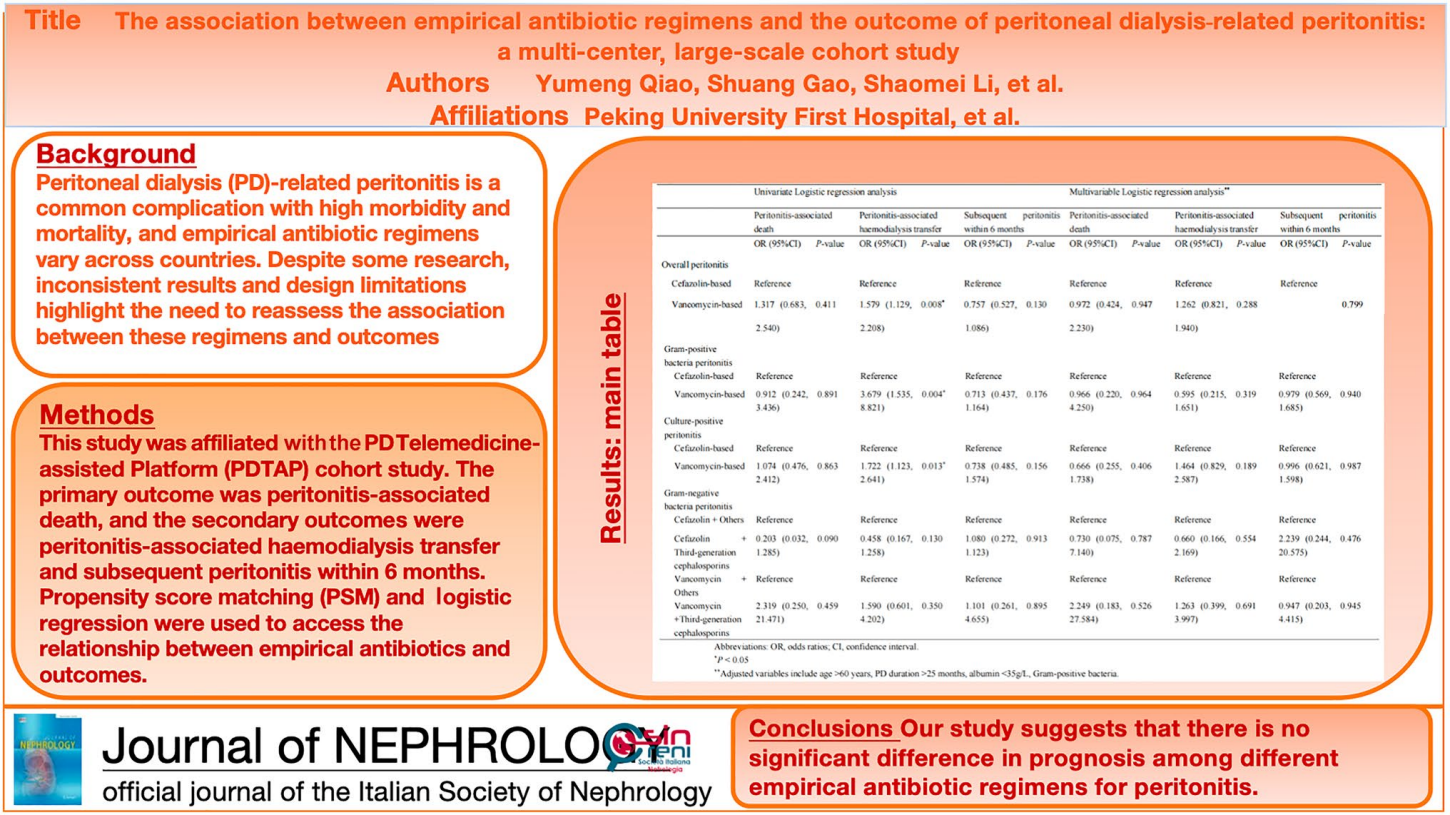

**Supplementary Information:**

The online version contains supplementary material available at 10.1007/s40620-025-02437-9.

## Introduction

Peritoneal dialysis- (PD) related peritonitis is a common complication among PD patients, resulting in a significant number of hospitalizations [1, 2], hemodialysis transfers [2–4] and mortality [2, 4] in this population. Traditional empirical antibiotic regimens should include antibiotics against Gram-positive bacteria, such as cefazolin and vancomycin, as well as those against Gram-negative bacteria, mainly including third-generation cephalosporins and aminoglycosides [5]. There are currently no antimicrobial regimens with absolute advantages in the improvement of the prognosis of peritonitis [5]. Therefore, empirical antibiotic regimens used in different countries around the world vary widely. According to the Peritoneal Dialysis Outcomes and Practice Patterns Study (PDOPPS) latest data [4], the frequency of using the same common antibiotic, such as vancomycin, as an empirical antibiotic varies from 10 to 90%.

While many studies have investigated the influence of different antibiotic regimens on the prognosis of peritonitis, the results remain inconsistent [4, 6–25]. Of note, most of these studies are hindered by limitations such as single-center designs, small sample sizes, and a lack of representativeness [9, 10, 12, 17, 22–24]. Additionally, the inconsistency in baseline characteristics between patients in different antibiotic groups could undermine the validity and reliability of the findings [6, 7, 10, 13, 20, 23, 24, 26, 27]. Finally, it is noteworthy that approximately two-thirds of existing research dates back more than a decade [11, 25]. Given the increasing emergence of specific pathogens and drug-resistant bacteria in recent years [28–31], a comprehensive reevaluation of the impact of empirical antibiotic regimens on peritonitis outcomes is urgently needed. Recently, statisticians and epidemiologists have come to see real-world research as very important for understanding health issues and evaluating public health measures [32]. Therefore, apart from more robust evidence from multi-center randomized controlled trials (RCTs), a real-word observational study in a large PD population would be useful to provide meaningful data.

Therefore, our aim was to investigate the association between empirical antibiotic regimens and clinical outcomes of peritonitis, specifically comparing cefazolin and vancomycin. We utilized a nationwide multi-center cohort of PD patients in China and employed propensity score matching to minimize the influence of confounding factors on prognosis.

## Methods

### Study cohort and follow up

The study was affiliated with the PD Telemedicine-assisted Platform Cohort Study (PDTAP study), a nationwide, large-scale PD cohort in China. Center enrollment, participant eligibility, and enrollment details can be found in our previous article [33]. We screened all incident PD patients between 1st June, 2016 and 30th April, 2019 who were 18–80 years of age and had undergone PD treatment for > 3 months due to kidney failure. Patients were followed up until death (recorded cause), hemodialysis transfer (recorded cause), kidney transplantation, loss to follow-up, or until the end of the study (December 31st, 2020). Informed consent was obtained from each subject.

During the follow-up period, we recorded the first episode of peritonitis and tracked the prognosis over 1 month, including clinical cure, hemodialysis transfer, or death. Patients were excluded if they were lost to follow-up during the episode of peritonitis or lacked relevant laboratory data.

The study was conducted in accordance with the Declaration of Helsinki and was approved by the ethics committee of Peking University First Hospital (2018-100).

### Study outcome

The primary outcome of this study was peritonitis-associated death, with a secondary outcome centered on peritonitis-associated hemodialysis transfer. An additional secondary outcome was the occurrence of subsequent peritonitis within 6 months.

1. Peritonitis-associated death: Death occurring within 30 days of peritonitis onset or death during hospitalization due to peritonitis [5].
2. Peritonitis-associated hemodialysis transfer: Transfer from PD to hemodialysis for any period as part of the treatment for peritonitis [5].
3. Subsequent peritonitis within 6 months: We defined this as the recurrence of peritonitis occurring within 6 months after the peritonitis episode, regardless of the causative organisms.

### Demographic and laboratory data

Baseline characteristics included age, gender, body mass index (BMI), annual income, diabetes mellitus (DM), cardiovascular disease (CVD), and previous history of peritonitis. Duration of PD before the onset of peritonitis was recorded. Laboratory tests, including baseline hemoglobin, serum levels of albumin, lipids, and electrolytes (calcium, phosphorus, potassium, and sodium), were measured. Laboratory tests were recorded as the most recent values within 6 months prior to the onset of peritonitis.

### Peritonitis-related measurements

Patients were instructed to bring the first cloudy fluid to the dialysis center immediately once peritonitis was suspected. Examination of the PD effluent included white blood cell (WBC) count, causative organisms, and drug sensitivity. Two aliquots of dialysis effluent were sent for WBC (5 ml into tubes) count and bacterial culture (10 ml in aerobic and anaerobic blood culture bottles, respectively) using the blood-culture bottle kits (e.g. BACTEC). For each episode of peritonitis, we recorded the initial WBC count in the dialysis effluent, and then the serial WBC count on scheduled follow-up visits depending on each center’s workflow. The WBC counts in dialysis effluent on days 1 and 3 of peritonitis were recorded for analysis in this study.

The diagnosis of peritonitis followed the standards of the ISPD [5], including: (1) clinical features consistent with peritonitis, i.e., abdominal pain and/or cloudy dialysis effluent; (2) dialysis effluent WBC > 100/mL or > 100*10^6^/L (after a dwell time of at least 2 h), with > 50% polymorphonuclear leukocytes; (3) positive dialysis effluent culture. At least 2 of these 3 items were sufficient for diagnosis.

### Empirical antibiotic regimen information

According to the ISPD peritonitis guideline, an empirical antibiotic regimen for peritonitis should provide adequate coverage for both Gram-positive and Gram-negative organisms [5]. In this study, empirical antibiotics are defined as the initial regimen prescribed within 3 days of peritonitis onset. Patients were divided into cefazolin-based and vancomycin-based groups based on the antibiotics used to cover Gram-positive bacteria. In each group, patients were further divided into third-generation cephalosporins and other antibiotics subgroups according to the antibiotics covering Gram-negative bacteria. We recorded the type and duration of each antibiotic administered during peritonitis treatment. Episodes of peritonitis with antibiotic monotherapy were excluded from the analysis. Information on the use of preventive antifungals was incomplete and thus not analyzed.

### Statistical analysis

Normal distribution data were presented as mean ± standard deviation and compared by independent samples t-test across groups. Non-normal distribution variables were presented as median (interquartile range (IQR)) or frequency (count) and compared across relevant groups using the Mann–Whitney *U* test for continuous variables and chi-square test or Fisher’s exact test for categorical variables.

Primary and secondary outcomes of peritonitis were first compared between the cefazolin-based and vancomycin-based groups among all episodes of peritonitis as well as episodes caused by Gram-positive bacteria. In addition, for patients with Gram-negative bacterial peritonitis, we further compared the differences in peritonitis prognosis among four subgroups: cefazolin plus third-generation cephalosporin, cefazolin plus other antibiotics, vancomycin plus third-generation cephalosporin, and vancomycin plus other antibiotics.

Univariate logistic regression analysis was then employed to explore the relationships between antibiotic regimens and primary and secondary outcomes of peritonitis by calculating odds ratios and 95% confidence intervals (CIs). Based on our preliminary findings, age > 60 years, albumin < 35 g/L, PD duration > 25 months, effluent WBC > 300/mm^3^ on day 3, and causative organisms were significantly associated with peritonitis outcome. These variables were subsequently considered as significant confounders in the multivariate logistic regression analysis to further determine the independent impact of empirical antibiotic regimens on peritonitis outcomes.

To further address differences between groups and eliminate potential factors affecting the prognosis of peritonitis, propensity score matching analyses were conducted on five risk factors along with other variables exhibiting differences between groups (annual income, CVD, DM, peritonitis history and hemoglobin between cefazolin-based and vancomycin-based groups for overall peritonitis; gender, annual income and CVD between cefazolin-based and vancomycin-based groups for Gram-positive peritonitis; annual income between third-generation cephalosporins and other antibiotics subgroups within cefazolin-based group for Gram-negative peritonitis; annual income and peritonitis history between third-generation cephalosporins and other antibiotic subgroups within the vancomycin-based group for Gram-negative peritonitis). Matching was performed at a 1:1 ratio, using nearest neighbor matching with a caliper width of 0.02.

Finally, to enhance the robustness of the study, after excluding cases of culture-negative peritonitis, we conducted a further sensitivity analysis on the relationship between empirical antibiotics and peritonitis outcomes in patients with culture-positive peritonitis. The propensity score matching variables included five risk factors, as well as gender, annual income, DM, peritonitis history, hemoglobin, calcium, and phosphorus.

Statistical analyses were performed using SPSS version 26.0 (IBM, Armonk, NY, USA) and R version 4.3.1 (R Foundation for Statistical Computing, Vienna, Austria). Two-tailed *P* < 0.05 findings were considered statistically significant, except where otherwise indicated.

## Results

### Study cohort and antibiotic regimens

**Fig. 1:**
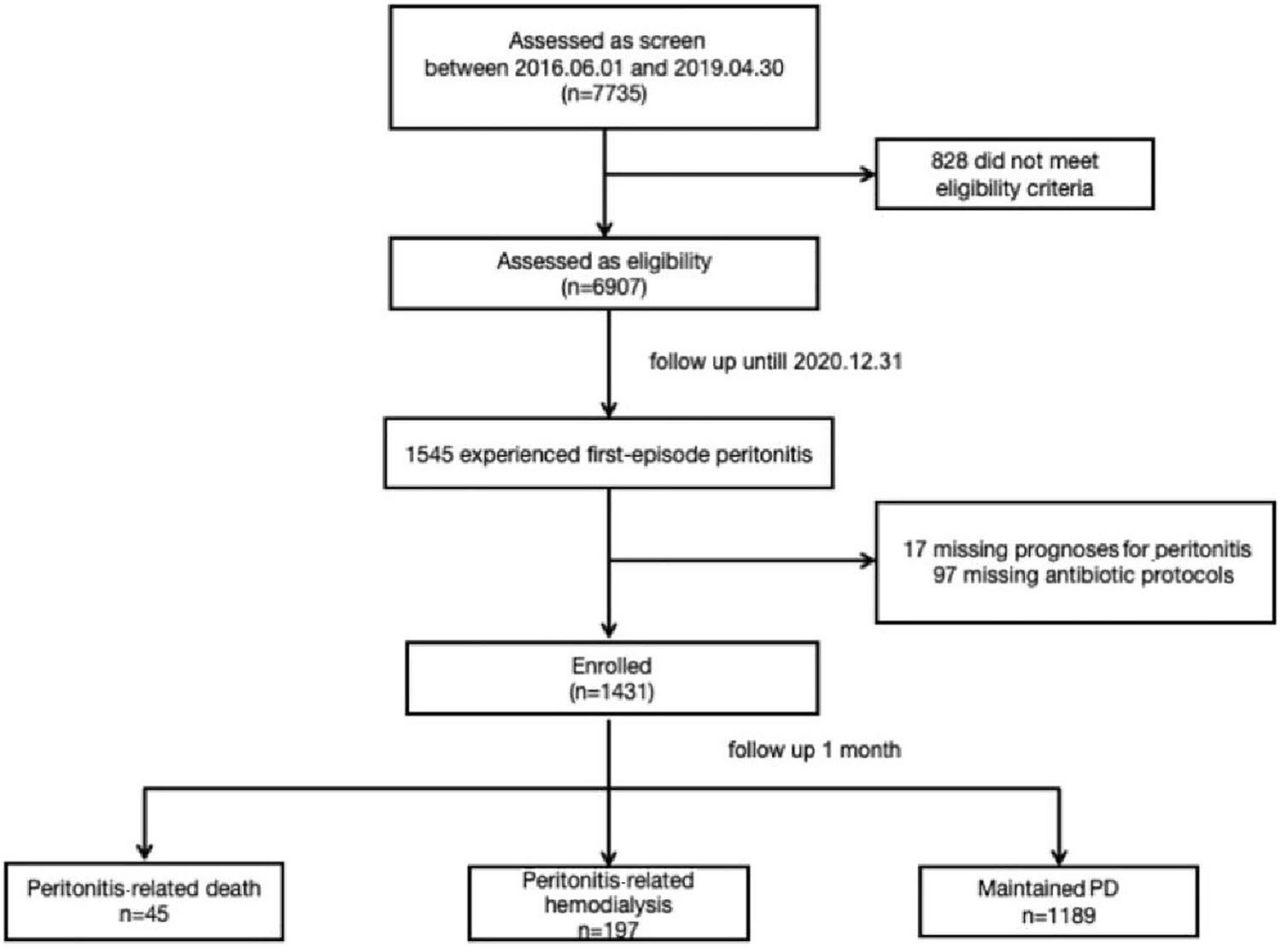
Flow chart of study cohort

From June 1st, 2016, to April 30th, 2019, 7735 patients in the PDTAP cohort were enrolled and followed up, and eventually, 1431 patients who experienced first-episode peritonitis and met the inclusion criteria were included in this study (Fig. 1). There were 803 males (56.12%), with a mean age of 52.66 ± 14.35 years and a median PD duration of 27.80 (10.33, 50.60) months. Forty-five patients died and 197 patients transferred to hemodialysis within 30 days of peritonitis onset, while 1189 patients clinically recovered and maintained PD.

The majority of patients with peritonitis (1203, 84.07%) were treated with either intraperitoneal vancomycin or cephazolin in combination with third-generation cephalosporins or other antibiotics, while a small number received monotherapy (132, 9.22%) or other regimens (96, 6.71%) (Supplement Fig. 1). There was no statistically significant difference in the effect of various antibiotic regimens on the prognosis of peritonitis (*P* = 0.299) (Supplement Fig. 2).

### Comparisons of clinical characteristics and laboratory data between the cefazolin-based group and the vancomycin-based group

The 1203 peritonitis patients were divided into the cefazolin-based group (*n* = 637) and the vancomycin-based group (*n* = 566) based on the antibiotics used against Gram-positive bacteria. Clinical characteristics and laboratory data are compared between groups (Table 1). Patients in the vancomycin-based group were older, had longer PD duration, and higher annual income (*P* < 0.05 for all). Additionally, this group had a higher proportion of DM, CVD, and peritonitis history, along with higher hemoglobin levels (*P* < 0.05 for all). Compared with those receiving cefazolin, patients treated with vancomycin as the empirical antibiotic had a higher number of effluent WBCs on day 3 (*P* < 0.001) (Table 2).

**Table 1:** Comparison of clinical characteristics between the cefazolin-based and vancomycin-based groups in overall patients

	Total (*n* = 1203)	Before matching			After matching		
		Cefazolin-based (*n* = 637)	Vancomycin-based (*n* = 566)	*P*-value	Cefazolin-based (*n* = 343)	Vancomycin-based (*n* = 343)	*P*-value
Clinical characteristics							
Age (years)	52.75 ± 14.27	50.91 ± 14.10	54.81 ± 14.18	< 0.001^*^	52.57 ± 14.17	53.25 ± 14.25	0.532
Age (> 60 years), *n* (%)	398 (33.1%)	179 (28.1%)	219 (38.7%)	< 0.001^*^	114 (33.24%)	112 (32.65%)	0.935
Male, *n* (%)	673 (55.9%)	371 (58.2%)	302 (53.4%)	0.100	196 (57.14%)	182 (53.06%)	0.318
BMI (kg/m2)	23.28 ± 3.60	23.19 ± 3.68	23.38 ± 3.52	0.373	23.27 ± 3.72	23.40 ± 3.45	0.643
PD duration (> 25 months)	651 (54.1%)	324 (50.9%)	327 (57.8%)	0.019^*^	185 (53.94%)	187 (54.52%)	0.939
PD duration (month)	28.50 (10.33, 52.11)	25.50 (10.27, 45.80)	31.15 (10.58, 58.98)	0.002^*^	28.23 (10.59, 46.35)	27.83 (9.84, 57.06)	0.280
Annual income, (> 7000$), *n* (%)	417 (34.66%)	194 (30.46%)	223 (39.40%)	0.001^*^	117 (34.11%)	123 (35.86%)	0.689
Cardiovascular disease, *n* (%)	424 (35.30%)	198 (31.08%)	226 (40.07%)	0.001^*^	126 (36.73%)	123 (35.86%)	0.874
Diabetes mellitus, *n* (%)	426 (35.41%)	200 (31.40%)	226 (39.93%)	0.002^*^	122 (35.57%)	131 (38.19%)	0.527
Peritonitis history, *n* (%)	221 (18.57%)	95 (15.10%)	126 (22.46%)	0.001^*^	64 (18.66%)	65 (18.95%)	1.000
Automated peritoneal dialysis, *n* (%)	24 (2.00%)	12 (1.88%)	12 (2.12%)	0.767	6 (1.75%)	7 (2.04%)	0.659
Laboratory variables							
Hemoglobin (g/L)	105.42 ± 20.16	103.80 ± 22.61	107.17 ± 16.96	0.005^*^	106.82 ± 21.57	105.74 ± 17.4	0.472
Serum albumin (g/L)	34.82 ± 5.52	34.82 ± 5.70	34.82 ± 5.32	0.990	35.33 ± 5.46	35.04 ± 5.24	0.480
Albumin (< 35 g/L), *n* (%)	516 (45.83%)	274 (46.84%)	242 (44.73%)	0.517	146 (42.57%)	151 (44.02%)	0.758
Triglycerides (mmol/L)	1.88 ± 1.43	1.81 ± 1.50	1.97 ± 1.33	0.085	2.02 ± 1.77	1.95 ± 1.26	0.592
Total cholesterol (mmol/L)	4.66 ± 1.31	4.61 ± 1.26	4.71 ± 1.37	0.255	4.71 ± 1.25	4.74 ± 1.39	0.723
Serum calcium (mmol/L)	2.21 ± 0.24	2.18 ± 0.25	2.24 ± 0.23	< 0.001^*^	2.21 ± 0.25	2.25 ± 0.22	0.026
Serum phosphorus (mmol/L)	1.60 ± 0.51	1.65 ± 0.55	1.55 ± 0.47	0.001^*^	1.61 ± 0.51	1.58 ± 0.49	0.402
Serum potassium (mmol/L)	4.14 ± 0.76	4.14 ± 0.77	4.13 ± 0.76	0.942	4.09 ± 0.77	4.20 ± 0.75	0.060
Serum sodium (mmol/L)	138.69 ± 5.72	138.94 ± 7.16	138.42 ± 3.62	0.132	138.43 ± 8.78	138.58 ± 3.34	0.769

*BMI* body mass index, *PD* peritoneal dialysis

^*^P < 0.05 or 0.001 between groups

**Table 2:** Comparison of peritonitis-related information and peritonitis outcome between the cefazolin-based and vancomycin-based groups in overall patients

	Total (*n* = 1203)	Before matching			After matching		
		Cefazolin-based (*n* = 637)	Vancomycin-based (*n* = 566)	*P*-value	Cefazolin-based (*n* = 343)	Vancomycin-based (*n* = 343)	*P*-value
Effluent WBC							
WBC count on day 1 (10^6^/L)	1453.00 (525.75, 3603.75)	1440.00 (470.00, 3662.00)	1500.00 (552.00, 3580.00)	0.316	1573.00 (554.00, 3885.00)	1530.00 (588.00, 3334.50)	0.965
WBC count on day 3 (10^6^/L)	100.00 (24.00, 393.75)	85.00 (19.00, 288.00)	124.00 (34.00, 545.00)	< 0.001^*^	94.00 (20.00, 372.50)	107.00 (33.00, 330.00)	0.227
WBC count on day 3 (> 300/mm^3^), *n* (%)	302 (29.41%)	127 (24.33%)	175 (34.65%)	< 0.001^*^	94 (27.41%)	93 (27.11%)	1.000
Causative organism, *n* (%)				0.302			0.771
Gram-positive	504 (44.52%)	271 (46.01%)	233 (42.91%)		157 (45.77%)	155 (45.19%)	
Gram-negative	230 (20.32%)	114 (19.35%)	116 (21.36%)		69 (20.12%)	66 (19.24%)	
Polymicrobial	18 (1.59%)	7 (1.19%)	11 (2.03%)		4 (1.17%)	7 (2.04%)	
Tuberculosis/Fungus	29 (2.56%)	11 (1.87%)	18 (3.31%)		7 (2.04%)	11 (3.21%)	
Culture-negative	351 (31.01%)	186 (31.58%)	165 (30.39%)		106 (30.90%)	104 (30.32%)	
Treatment failure, *n* (%)	198 (16.46%)	86 (13.50%)	112 (19.79%)	0.003^*^	47 (13.70%)	56 (16.33%)	0.336
Peritonitis-associated death	37 (3.11%)	17 (2.72%)	20 (3.55%)	0.514	8 (2.33%)	11 (3.21%)	0.642
Peritonitis-associated to hemodialysis transfer	161 (13.52%)	69 (11.00%)	92 (16.31%)	0.010^*^	39 (11.37%)	45 (13.12%)	0.560
Subsequent peritonitis within 6 months	137 (11.39%)	81 (12.72%)	56 (9.89%)	0.129	36 (10.50%)	38 (11.08%)	0.865

*PD* peritoneal dialysis, *WBC* white blood cell

^*^P < 0.05 or 0.001 between groups

Among patients with Gram-positive bacterial peritonitis, 504 patients were classified into cefazolin-based (*n* = 271) and vancomycin-based (*n* = 233) groups. Consistent with the above results, the vancomycin-based group was older, had a longer PD duration, and higher proportions of DM and high-income status, along with higher effluent WBC count on day 3 (*P* < 0.05 for all) (Supplement Table 1).

### Comparisons in peritonitis outcomes between the cefazolin-based group and the vancomycin-based group

#### Peritonitis-associated death

The probability of peritonitis-associated death was similar between the two groups for overall peritonitis (2.72% vs. 3.55%, *P* = 0.514), episodes caused by Gram-positive bacteria (1.88% vs. 1.72%, *P* = 1.000), and culture-negative peritonitis (2.56% vs. 1.61%, *P* = 0.231).

Univariate logistic regression indicated that empirical antibiotic treatment with vancomycin and cefazolin was not significantly associated with the likelihood of peritonitis-associated death in overall peritonitis episodes, as well as in those with Gram-positive bacterial peritonitis and culture-positive peritonitis (*P* > 0.05 for all) (Table 3). After adjusting for confounding factors, no significant difference was found between the cefazolin-based group and the vancomycin-based group (*P* > 0.05 for all).

**Table 3:** Odds ratios for peritonitis-associated death, hemodialysis transfer and subsequent peritonitis within 6 months by antibiotic regimens

		Univariate Logistic regression analysis						Multivariable Logistic regression analysis					
		Peritonitis-associated death		Peritonitis-associated hemodialysis transfer		Subsequent peritonitis within 6 months		Peritonitis-associated death		Peritonitis-associated hemodialysis transfer		Subsequent peritonitis within 6 months	
		OR (95%CI)	*P*-value	OR (95%CI)	*P*-value	OR (95%CI)	*P*-value	OR (95%CI)	*P*-value	OR (95%CI)	*P*-value	OR (95%CI)	*P*-value
Overall peritonitis													
Cefazolin-based		Reference		Reference		Reference		Reference		Reference		Reference	
Vancomycin-based		1.317 (0.683, 2.540)	0.411	1.579 (1.129, 2.208)	0.008^*^	0.757 (0.527, 1.086)	0.130	0.972 (0.424, 2.230)	0.947	1.262 (0.821, 1.940)	0.288		0.799
Gram-positive bacteria peritonitis													
Cefazolin-based		Reference		Reference		Reference		Reference		Reference		Reference	
Vancomycin-based		0.912 (0.242, 3.436)	0.891	3.679 (1.535, 8.821)	0.004^*^	0.713 (0.437, 1.164)	0.176	0.966 (0.220, 4.250)	0.964	0.595 (0.215, 1.651)	0.319	0.979 (0.569, 1.685)	0.940
Culture-positive peritonitis													
Cefazolin-based		Reference		Reference		Reference		Reference		Reference		Reference	
Vancomycin-based		1.074 (0.476, 2.412)	0.863	1.722 (1.123, 2.641)	0.013^*^	0.738 (0.485, 1.574)	0.156	0.666 (0.255, 1.738)	0.406	1.464 (0.829, 2.587)	0.189	0.996 (0.621, 1.598)	0.987
Gram-negative bacteria peritonitis													
Cefazolin + Others		Reference		Reference		Reference		Reference		Reference		Reference	
Cefazolin + Third-generation cephalosporins		0.203 (0.032, 1.285)	0.090	0.458 (0.167, 1.258)	0.130	1.080 (0.272, 1.123)	0.913	0.730 (0.075, 7.140)	0.787	0.660 (0.166, 2.169)	0.554	2.239 (0.244, 20.575)	0.476
Vancomycin + Others		Reference		Reference		Reference		Reference		Reference		Reference	
Vancomycin + Third-generation cephalosporins		2.319 (0.250, 21.471)	0.459	1.590 (0.601, 4.202)	0.350	1.101 (0.261, 4.655)	0.895	2.249 (0.183, 27.584)	0.526	1.263 (0.399, 3.997)	0.691	0.947 (0.203, 4.415)	0.945

*OR* odds ratios, *CI* confidence interval

^*^P < 0.05

^**^Adjusted variables include age > 60 years, PD duration > 25 months, albumin < 35 g/L, Gram-positive bacteria

To eliminate imbalances in prognostic factors and clinical characteristics between the groups, we conducted propensity score matching analysis. After matching, there were 343 pairs for overall peritonitis episodes, 142 pairs for Gram-positive peritonitis episodes, and 198 pairs for culture-positive peritonitis episodes (Table 2, Supplementary Tables 1 and 2). Subsequent analyses revealed no significant differences in the rates of peritonitis-associated death between the vancomycin-based and cefazolin-based groups for overall episodes, Gram-positive episodes, and culture-positive episodes (*P* > 0.05 for all) (Table 2 and Supplementary Tables 1 and 2).

#### Peritonitis-associated hemodialysis transfer

Compared to those receiving cefazolin, patients treated with vancomycin as the empirical antibiotic were more likely to require peritonitis-associated hemodialysis transfer. This was observed across different types of peritonitis: overall peritonitis (11.00% vs 16.31%, *P* = 0.010) (Table 2), peritonitis caused by Gram-positive bacteria (2.62% vs 9.01%, *P* = 0.004) (Supplement Table 1), and culture-positive peritonitis (15.92% vs 9.90%, *P* = 0.016) (Supplement Table 2).

Univariate logistic regression indicated that patients treated with vancomycin had a higher risk of peritonitis-associated hemodialysis transfer [OR 1.579 (1.129, 2.208), *P* = 0.008 in overall episodes; OR 3.679 (1.535, 8.821), *P* = 0.004 in Gram-positive cases; OR 1.722 (1.123, 2.641), *P* = 0.013 in culture-positive cases]. However, after adjustments for confounding factors, neither antibiotic was statistically associated with the risk of peritonitis-associated hemodialysis transfer (*P* > 0.05 for all).

After propensity score matching analyses, there were no significant differences in the rates of peritonitis-associated hemodialysis transfer between the vancomycin-based and cefazolin-based groups for overall episodes, episodes caused by Gram-positive bacteria, or culture-positive peritonitis (*P* > 0.05 for all) (Table 2 and Supplement Tables 1 and 2).

#### Subsequent peritonitis within 6 months

The probability of subsequent peritonitis within 6 months was similar between the two groups for overall peritonitis (12.72% vs 9.89%, *P* = 0.129), episodes caused by Gram-positive bacteria (17.71% vs 13.34%, *P* = 0.175) and culture-positive peritonitis (14.85% vs 11.41%, *P* = 0.188) (Table 2 and Supplement Tables 1 and 2). Similarly, both univariate logistic regression and multivariate logistic regression after adjusting for confounding factors indicated that empirical antibiotics, including vancomycin and cefazolin, were not significantly associated with the likelihood of subsequent peritonitis within 6 months (*P* > 0.05 for all) (Table 3).

### Comparison of third-generation cephalosporin and non-third-generation cephalosporin among episodes caused by Gram-negative bacteria

Among the 114 patients with Gram-negative bacterial peritonitis, 30 patients were treated with third-generation cephalosporins and 84 patients were treated with other antibiotics (both within the cefazolin-based regimen). Except for income and serum potassium levels, there were no significant differences in general clinical characteristics and laboratory tests between these two subgroups (Supplement Table 3). The rates of peritonitis-associated death, hemodialysis transfer, and subsequent peritonitis were similar. However, the composite outcome of peritonitis-related death and hemodialysis transfer was lower with cefazolin combined with third-generation cephalosporins compared to cefazolin combined with other antibiotics (17.86% vs. 36.67%, *P* = 0.035). Multivariate logistic regression analysis showed that third-generation cephalosporins were not significantly associated with the prognosis of Gram-negative bacterial peritonitis (*P* > 0.05 for all) (Table 3), and after propensity score matching for relevant confounding factors, no significant differences in clinical outcomes of peritonitis were observed between the two subgroups (*P* > 0.05 for all) (Supplement Table 3).

In the vancomycin-based group, 116 patients with Gram-negative bacterial peritonitis were treated with either third-generation cephalosporins (*n* = 75) or other antibiotics (*n* = 41). Patients who received third-generation cephalosporins were older, had longer PD duration, higher income, and were more likely to have a prior history of peritonitis (*P* < 0.05 for all) (Supplement Table 4). Despite these differences, the prognosis of peritonitis was comparable between the two subgroups (*P* > 0.05 for all). Similarly, univariate, and multivariate logistic regression analyses did not show any differences in the risk of peritonitis-associated death, hemodialysis transfer and subsequent peritonitis between subgroups (*P* > 0.05 for all) (Table 3). Additionally, propensity score matching analysis did not change the findings (Supplement Table 4).

## Discussion

In this study, we used a multi-center cohort to explore the association between empirical antibiotic regimens and the prognosis of peritonitis. To control for confounding factors, we employed propensity score matching to balance variables affecting the prognosis of peritonitis and those that differed between antibiotic groups. After matching, the results indicated that the use of empirical antibiotics—cefazolin or vancomycin—showed similar outcomes for overall peritonitis, Gram-positive bacterial peritonitis, and culture-negative peritonitis. In cases of Gram-negative bacterial peritonitis, the use of third-generation cephalosporins also had no association with better prognosis, irrespective of propensity score matching analysis (*P* > 0.05 for all).

The efficacy of empirical cefazolin or vancomycin has been investigated in recent years, mostly based on analyses from observational studies. Consistent with our findings, the PDOPPS study recently indicated no significant difference in the prognosis for peritonitis between empirical cefazolin and vancomycin [4]. However, other studies showed inconsistent findings [7–13, 23, 25]. Several potential causes of inconsistent conclusions among studies are listed below. First, basic characteristics of participants such as age, body size, serum albumin level and residual kidney function and antibiotics regimen differed across these studies, which could have impacted the effective blood concentration of vancomycin [34, 35]. As reported, patients whose nadir vancomycin level exceeded 15 mg/L increased the likelihood of clinical cure by 7.5 times compared to those who did not achieve this level [36]. Second, the rates of methicillin-resistant strains might have varied by center [6, 8, 23, 29]. In centers with a high incidence of methicillin-resistant bacteria, the empirical use of vancomycin should be considered, as it may improve outcome [5]. Third, potential confounders for peritonitis outcomes such as PD duration, causative organism and effluent WBC between antibiotic treatments potentially introduced bias thus compromising the reliability of the findings [4, 13, 37, 38].

In our study, univariate logistic regression indicates that patients receiving vancomycin as empirical antibiotic therapy have a higher risk of transfer to hemodialysis. This finding may be attributed to the older age, longer PD duration, greater comorbidity, and higher effluent WBC count among patients in the vancomycin group. This phenomenon aligns with the tendency of clinicians to empirically use vancomycin for patients with more severe conditions. Further, after adjusting for relevant confounding factors or conducting propensity score matching analysis, the effects of vancomycin and cefazolin on the prognosis of peritonitis appear to be comparable. These findings support our hypothesis on the confounding effect in the association of antibiotics regimen and peritonitis outcomes in previous studies. Based on the current findings, we do not recommend the use of vancomycin as empirical therapy to enhance efficacy. Excessive vancomycin use could lead to the emergence of vancomycin-resistant Staphylococcus aureus (VRSA) or vancomycin-resistant enterococci (VRE), with increasing prevalence over time [31, 39, 40]. This resistance complicates treatment and worsens outcomes for peritonitis patients [41]. Furthermore, due to its unique pharmacokinetics, serum vancomycin concentration significantly impacts the efficacy of treatment for peritonitis, adding complexity and expense to clinical management [34, 36].

The incidence of Gram-negative bacterial peritonitis ranges from 10 to 30% and has been increasing in recent years, typically associated with a poor prognosis [1, 4, 37, 42]. Therefore, selecting the appropriate empirical treatment is crucial. By utilizing propensity score matching analysis, our study demonstrated that third-generation cephalosporins have a comparable effect on prognosis as other antibiotics against Gram-negative bacteria. However, the limited number of cases and diverse antibiotics options hinder the identification of any specific antibiotic linked to improved outcomes. Current literature presents inconsistent findings regarding the efficacy of antibiotics against Gram-negative bacteria, with no clear superior option [12, 16, 17, 19, 20, 25, 26]. Compared to ciprofloxacin, empirical ceftazidime resulted in fewer recurrences and technical failures, but did not significantly outperform gentamicin [20]. A meta-analysis found that initial treatment with glycopeptides plus ceftazidime led to a higher clinical cure rate than glycopeptides plus aminoglycosides [25], while the PDOPPS study indicated gentamicin was more effective than ceftazidime [4]. Some RCTs also found no significant differences in peritonitis outcomes among third-generation cephalosporins, aminoglycosides, and fluoroquinolones in empirical treatment regimens [17, 19]. Given the small sample sizes and limited evidence from current studies, future larger cohort studies or RCTs are necessary to investigate the effect of different antibiotics on the prognosis of Gram-negative bacterial peritonitis.

This is the first study using propensity score matching analysis, which could mimic RCTs studies [43], to examine the effects of antibiotics on peritonitis prognosis. By controlling for confounding factors, we have mitigated biases and enhanced the robustness and credibility of our conclusions, providing valuable insights into the comparable effectiveness of different empirical antibiotics. By utilizing a nationwide PD cohort representing a diverse sample from seven geographical regions, our study enhances the reliability and generalizability of the findings. This makes the results applicable to a broad population of PD patients and relevant to clinical practices across various settings. The cohort includes peritonitis cases from 2016 to 2020, providing the most recent multicenter data on the impact of antibiotics on peritonitis outcomes. Additionally, the treatment failure rates for Gram-positive and Gram-negative bacterial peritonitis observed in this study align with those of previous studies, further supporting the representativeness of the sample [37, 42, 44, 45].We also performed separate analyses on the efficacy of different antibiotics for overall peritonitis, as well as for Gram-positive and Gram-negative bacterial peritonitis, enhancing the applicability and generalizability of our findings. We focused solely on first-episode peritonitis cases to avoid repeated analyses of individual cases, thereby minimizing observer bias. Our follow-up for the cohort is largely complete, with comprehensive baseline clinical characteristics, biochemical parameters, and peritonitis-related information. The overall missing case rate is just 7.38%, with only 1.10% lost to follow-up, which is lower than that observed in other large-scale cohort studies [46].

We are also aware of the limitations of this study. First, we could not completely rule out the impact of researcher bias on the results due to the characteristics of observational studies. Inevitably, different clinicians tend to favor varying antibiotic regimens when dealing with patients presenting with different conditions. Moreover, even with the use of propensity score matching analysis, we could not fully eliminate all confounding factors. Although the sensitivity analysis excluding culture-negative peritonitis cases did not alter the study results, the high proportion of up to 30% culture-negative cases remains a limitation of our study. We focused exclusively on the types of empirical antibiotics and did not include the duration of antibiotic treatment or subsequent adjustments based on sensitivity results in our analysis. Lastly, these data may not be generalizable to other countries with different populations and PD practices.

## Conclusion

In summary, our nationwide PD cohort study indicates that various empirical antibiotic regimens for treating peritonitis do not differ significantly with regard to the rate of peritonitis-associated death or hemodialysis transfer and subsequent peritonitis within 6 months. The study found no association between the empirical use of vancomycin and improved clinical outcomes in peritonitis. Although propensity score matching analyses enhance the credibility of our conclusions, more robust evidence from multicenter interventional trials is still needed to guide empiric antibiotics regimens in order to improve peritonitis outcomes.

## Supplementary Information

Below is the link to the electronic supplementary material.

Supplementary file1 (DOCX 282 KB)

## Data Availability

Data described in the manuscript, code book, and analytic code will not be made available because the Management of China’s Human Genetic Resources does not allow sharing of the information.
