# Supplementary material for: The association between empirical antibiotic regimens and the outcome of peritoneal dialysis-related peritonitis: a multi-center, large-scale cohort study
Source: J Nephrol. 2025 Oct 24;38(9):2673–85. doi: 10.1007/s40620-025-02437-9 (PMC12712009; doi:10.1007/s40620-025-02437-9)
Supplement: Supplementary file 1 — Supplementary file1 (DOCX 282 KB) [file 40620_2025_2437_MOESM1_ESM.docx]

**Supplement Table 1. Comparison of clinical characteristics and peritonitis related information between the cefazolin-based and vancomycin-based groups in Gram-positive bacterial peritonitis.**

|  | |  | Before matching | | | After matching | | |
| --- | --- | --- | --- | --- | --- | --- | --- | --- |
|  | | Total  (n=504) | Cefazolin-based  (n=271) | Vancomycin-based  (n=233) | *P*-value | Cefazolin-based  (n=142) | Vancomycin-based  (n=142) | *P-*value |
| Clinical characteristics | |  |  |  |  |  |  |  |
|  | Age (years) | 52.76 ± 13.76 | 51.04 ± 13.51 | 54.75 ±13.81 | 0.002^*^ | 54.52 ± 14.06 | 55.16 ± 13.37 | 0.436 |
|  | Age (>60 years), n (%) | 158 (31.3%) | 77 (28.4%) | 81 (34.8%) | 0.151 | 55 (34.59%) | 58 (36.48%) | 1.000 |
|  | Male, n (%) | 322 (63.89%) | 187 (69.00%) | 135 (57.94%) | 0.013^*^ | 94 (66.20%) | 84 (59.15%) | 0.270 |
|  | BMI (kg/m2) | 23.54 ± 3.62 | 23.39 ± 3.61 | 23.73 ± 3.63 | 0.292 | 23.35 ± 3.59 | 23.56 ± 3.56 | 0.629 |
|  | PD duration (>25 months), n (%) | 273 (54.17%) | 139 (51.29%) | 134 (57.51%) | 0.191 | 79 (55.63%) | 84 (59.15%) | 0.631 |
|  | PD duration (month) | 28.30 (9.93, 48.63) | 26.50 (8.66, 41.97) | 30.50 (11.10, 57.60) | 0.006^*^ | 28.30 (8.27, 46.02) | 30.44 (11.20, 64.05) | 0.053 |
|  | Annual income, (>50,000¥), n (%) | 175 (34.72%) | 82 (30.26%) | 93 (39.91%) | 0.030^*^ | 50 (36.62%) | 48 (33.80%) | 0.901 |
|  | Cardiovascular disease, n (%) | 166 (32.94%) | 75 (27.68%) | 91 (39.06%) | 0.009^*^ | 52 (36.62%) | 45 (31.69%) | 0.453 |
|  | Diabetes mellitus, n (%) | 178 (35.32%) | 88 (32.47%) | 90 (38.63%) | 0.178 | 43 (30.28%) | 53 (37.32%) | 0.259 |
|  | Peritonitis history, n (%) | 86 (17.06%) | 38 (14.02%) | 48 (20.60%) | 0.066 | 22 (15.49%) | 30 (21.13%) | 0.283 |
|  | Automated peritoneal dialysis, n (%) | 7 (1.39%) | 3 (1.11%) | 4 (1.72%) | 0.709 | 2 (1.41%) | 1 (0.70%) | 0.522 |
| Laboratory variables | |  |  |  |  |  |  |  |
|  | Hemoglobin (g/L) | 105.19 ± 20.00 | 104.02 ± 22.31 | 106.55 ± 16.86 | 0.165 | 105.76 ± 20.68 | 106.86 ± 15.96 | 0.620 |
|  | Serum albumin (g/L) | 34.82 ± 5.51 | 34.66 ± 5.87 | 35.00 ± 5.06 | 0.500 | 35.22 ± 5.60 | 34.77 ± 5.23 | 0.490 |
|  | Albumin (<35g/L), n (%) | 217 (45.78%) | 126 (49.61%) | 91 (41.36%) | 0.088 | 62 (43.66%) | 62 (43.66%) | 1.000 |
|  | Triglyceride (mmol/L) | 1.78 ± 1.23 | 1.70 ± 1.33 | 1.89 ± 1.10 | 0.123 | 1.69 ± 1.12 | 1.89 ± 1.25 | 0.208 |
|  | Total cholesterol (mmol/L) | 4.55 ± 1.19 | 4.52 ± 1.20 | 4.59 ± 1.17 | 0.571 | 4.75 ± 1.17 | 4.56 ± 1.28 | 0.225 |
|  | Serum calcium (mmol/L) | 2.21 ± 0.23 | 2.17 ± 0.23 | 2.25 ± 0.22 | 0.001^*^ | 2.20 ± 0.22 | 2.24 ± 0.21 | 0.089 |
|  | Serum phosphorus (mmol/L) | 1.62 ± 0.54 | 1.65 ± 0.59 | 1.58 ± 0.48 | 0.138 | 1.63 ± 0.54 | 1.54 ± 0.48 | 0.144 |
|  | Serum potassium (mmol/L) | 4.16 ± 0.80 | 4.13 ± 0.78 | 4.19 ± 0.82 | 0.402 | 4.12 ± 0.70 | 4.13 ± 0.74 | 0.881 |
|  | Serum sodium (mmol/L) | 138.40 ± 7.24 | 138.66 ± 9.41 | 138.09 ± 3.44 | 0.382 | 138.12 ± 12.12 | 138.11 ± 3.49 | 0.993 |
| Effluent WBC | |  |  |  |  |  |  |  |
|  | WBC on day 1 (10^^6^/L) | 1447.50 (600.00，3407.50) | 1531.00 (528.50, 3601.00) | 1376.00 (645.00, 2990.00) | 0.945 | 1685.00 (711.00, 3417.50) | 1290.00 (676.50, 27457.00) | 0.177 |
|  | WBC on day 3 (10^^6^/L) | 70.00 (20.00, 220.00) | 58.00 (13.00, 198.50) | 83.00 (31.00, 287.50) | 0.005^*^ | 65.00 (16.25, 201.50) | 62.00 (24.25, 155.75) | 0.693 |
|  | WBC on day 3 (>300/mm^3^), n (%) | 93 (20.9%) | 39 (17.0%) | 54 (25.0%) | 0.048^*^ | 24 (16.90%) | 24 (16.90%) | 1.000 |
| Treatment failure, n (%) | | 37 (7.34%) | 12 (4.43%) | 25 (10.73%) | 0.007^*^ | 8 (5.63%) | 13 (9.15%) | 0.257 |
|  | Peritonitis-associated death | 9 (1.80%) | 5 (1.88%) | 4 (1.72%) | 1.000 | 4 (2.82%) | 4 (2.82%) | 1.000 |
|  | Peritonitis-associated to haemodialysis transfer | 28 (5.60%) | 7 (2.62%) | 21 (9.01%) | 0.004^*^ | 4 (2.82%) | 9 (6.34%) | 0.256 |
| Subsequent peritonitis within 6 months | | 79 (15.67%) | 48 (17.71%) | 31 (13.34%) | 0.175 | 20 (14.08%) | 21 (14.79%) | 0.972 |

Abbreviations: BMI, body mass index; PD, peritoneal dialysis; WCC, white cell counts.

^*^*P* < 0.05 or 0.001 between groups

**Supplement Table 2. Comparison of clinical characteristics and peritonitis related information between the cefazolin-based and vancomycin-based groups in culture-positive peritonitis.**

|  |  |  | Before matching | | | After matching | | |
| --- | --- | --- | --- | --- | --- | --- | --- | --- |
|  |  | Total  (n=852) | Cefazolin-based  (n=638) | Vancomycin-based  (n=565) | *P*-value | Cefazolin-based  (n=198) | Vancomycin-based  (n=198) | *P*-value |
| Clinical characteristics | |  |  |  |  |  |  |  |
|  | Age (years) | 53.69 ± 14.09 | 51.89 ± 14.01 | 55.62 ± 13.94 | <0.001^*^ | 53.28 ± 14.04 | 54.42 ± 14.27 | 0.427 |
|  | Age (>60 years), n (%) | 271 (34.70%) | 119 (29.46%) | 152 (40.32%) | 0.002^*^ | 69 (34.85%) | 76 (38.38%) | 0.531 |
|  | Male, n (%) | 450 (57.62%) | 252 (62.38%) | 198 (52.52%) | 0.007^*^ | 118 (59.60%) | 127 (64.14%) | 0.408 |
|  | BMI (kg/m2) | 23.46 ± 3.50 | 23.31 ± 3.50 | 23.62 ± 3.49 | 0.223 | 23.25 ± 3.36 | 23.82 ± 3.54 | 0.105 |
|  | PD duration (month) | 29.23 (11.43, 50.73) | 26.76 (20.49, 44.53) | 31.23 (12.30, 58.90) | 0.001^*^ | 125.85 (11.23, 43.43) | 29.02 (10.98, 51.20) | 0.203 |
|  | PD duration (>25 months), n (%) | 436 (55.83%) | 208 (51.49%) | 228 (60.48%) | 0.014^*^ | 100 (50.51%) | 112 (56.57%) | 0.268 |
|  | Annual income, (>50,000¥), n (%) | 283 (36.33%) | 124 (30.69%) | 159 (42.40%) | 0.001^*^ | 69 (34.85%) | 73 (36.87%) | 0.753 |
|  | Cardiovascular disease, n (%) | 277 (35.47%) | 131 (32.43%) | 146 (38.73%) | 0.078 | 67 (33.84%) | 72 (36.36%) | 0.674 |
|  | Diabetes mellitus, n (%) | 283 (36.24%) | 127 (31.44%) | 156 (41.38%) | 0.005^*^ | 70 (35.35%) | 75 (37.88%) | 0.677 |
|  | Peritonitis history, n (%) | 147 (18.87%) | 61 (15.17%) | 86 (22.81%) | 0.009^*^ | 34 (17.17%) | 40 (20.20%) | 0.519 |
| Laboratory variables | |  |  |  |  |  |  |  |
|  | Hemoglobin (g/L) | 105.51 ± 19.36 | 103.80 ± 21.45 | 107.33 ± 16.70 | 0.013^*^ | 106.94 ± 20.29 | 106.18 ± 16.97 | 0.685 |
|  | Serum albumin (g/L) | 34.85 ± 5.45 | 34.70 ± 5.80 | 35.01 ± 5.06 | 0.431 | 35.32 ± 5.44 | 34.92 ± 5.0 | 0.465 |
|  | Albumin (<35g/L), n (%) | 332 (45.29%) | 181 (48.40%) | 151 (42.06%) | 0.099 | 85 (42.93%) | 85 (42.93%) | 1.000 |
|  | Triglyceride (mmol/L) | 1.89 ± 1.42 | 1.84 ± 1.54 | 1.96 ± 1.25 | 0.291 | 2.08 ± 1.77 | 1.81 ± 1.13 | 0.103 |
|  | Total cholesterol (mmol/L) | 4.60 ± 1.27 | 4.58 ± 1.30 | 4.62 ± 1.25 | 0.693 | 4.70 ± 1.24 | 4.62 ± 1.30 | 0.567 |
|  | Serum calcium (mmol/L) | 2.22 ± 0.23 | 2.18 ± 0.23 | 2.25 ± 0.21 | <0.001^*^ | 2.23 ± 0.23 | 2.23 ± 0.20 | 0.983 |
|  | Serum phosphorus (mmol/L) | 1.60 ± 0.52 | 1.64 ± 0.56 | 1.56 ± 0.48 | 0.035^*^ | 1.56 ± 0.57 | 1.57 ± 0.50 | 0.867 |
|  | Serum potassium (mmol/L) | 4.14 ± 0.78 | 4.12 ± 0.78 | 4.16 ± 0.78 | 0.461 | 4.01 ± 0.71 | 4.18 ± 0.69 | 0.019 |
|  | Serum sodium (mmol/L) | 138.35 ± 6.54 | 138.60 ± 8.48 | 138.11 ± 3.65 | 0.320 | 138.19 ± 5.17 | 138.31 ± 3.54 | 0.887 |
| Effluent WBC | |  |  |  |  |  |  |  |
|  | WBC on day 1 (10^^6^/L) | 1749.00 (639.50, 4490.00) | 1751.00 (609.25, 4387.50) | 1749.00 (723.00, 4500.00) | 0.677 | 2213.00 (687.00, 5512.75 | 1511.50 (639.25, 4175.50) | 0.142 |
|  | WBC on day 3 (10^^6^/L) | 110.00 (29.00, 497.00) | 86.00 (20.00, 310.25) | 155.00 (40.00, 721.50) | <0.001^*^ | 100.00 (20.50, 472.25) | 99.00 (34.00, 394.00) | 0.634 |
|  | WBC on day 3 (>300/mm^3^), n (%) | 224 (31.95%) | 87 (24.93%) | 137 (38.92%) | <0.001^*^ | 62 (31.31%) | 56 (28.28%) | 0.583 |
| Treatment failure | | 124 (15.88%) | 52 (12.87%) | 72 (19.10%) | 0.023^*^ | 26 (13.13%) | 32 (16.16%) | 0.477 |
|  | Peritonitis-associated death | 24 (3.07%) | 12 (2.97%) | 12 (3.18%) | 1.000 | 4 (2.02%) | 7 (3.54%) | 0.541 |
|  | Peritonitis-associated to haemodialysis transfer | 100 (12.80%) | 40 (9.90%) | 60 (15.92%) | 0.016^*^ | 22 (11.11%) | 25 (12.63%) | 0.756 |
| Subsequent peritonitis within 6 months | | 103 (13.19%) | 60 (14.85%) | 43 (11.41%) | 0.188 | 31 (15.66%) | 26 (13.13%) | 0.567 |

Abbreviations: BMI, body mass index; PD, peritoneal dialysis; WCC, white cell counts.

^*^*P* < 0.05 or 0.001 between groups

**Supplement Table 3. Comparison of clinical characteristics and peritonitis related information between third-generation cephalosporins and other antibiotics for Gram-negative bacterial peritonitis in the cefazolin-based subgroup**

|  | |  | Before matching | | | After matching | | |
| --- | --- | --- | --- | --- | --- | --- | --- | --- |
|  | | Total  (n=114) | Other antibiotics  (n=30) | Third-generation cephalosporin  (n=84) | *P*-value | Other antibiotics  (n=15) | Third-generation cephalosporin  (n=15) | *P*-value |
| Clinical characteristics | |  |  |  |  |  |  |  |
|  | Age (years) | 53.54 ± 14.84 | 55.00 ± 15.80 | 53.02 ± 14.54 | 0.550 | 54.26 ± 15.09 | 52.56 ± 14.01 | 0.752 |
|  | Age (>60 years), n (%) | 35 (30.70%) | 10 (33.33%) | 25 (29.76%) | 0.894 | 4 (26.67%) | 4 (26.67%) | 1.000 |
|  | Male, n (%) | 55 (48.25%) | 12 (40.00%) | 43 (51.19%) | 0.401 | 7 (46.67%) | 9 (60.00%) | 0.714 |
|  | BMI (kg/m2) | 23.14 ± 3.28 | 23.10 ± 3.39 | 23.15 ± 3.26 | 0.947 | 23.09 ± 2.00 | 23.45 ± 2.87 | 0.690 |
|  | PD duration (month) | 24.90 (12.24, 45.57) | 39.92 (21.20, 63.16) | 22.63 (11.10, 40.64) | 0.006^*^ | 45.47 (14.68, 75.85) | 31.40 (11.52, 54.65) | 0.395 |
|  | PD duration (>25 months), n (%) | 56 (49.1%) | 19 (63.33%) | 37 (44.05%) | 0.109 | 9 (60.00%) | 9 (60.00%) | 1.000 |
|  | Annual income, (>50,000¥), n (%) | 32 (28.07%) | 15 (50.00%) | 17 (20.24%) | 0.004^*^ | 6 (40.00%) | 6 (40.00%) | 1.000 |
|  | Cardiovascular disease, n (%) | 49 (42.98%) | 17 (56.67%) | 32 (38.10%) | 0.121 | 8 (53.33%) | 7 (46.67%) | 1.000 |
|  | Diabetes mellitus, n (%) | 34 (29.82%) | 8 (26.67%) | 26 (30.95%) | 0.835 | 4 (26.67%) | 6 (40.00%) | 0.699 |
|  | Peritonitis history, n (%) | 18 (16.07%) | 7 (24.14%) | 11 (13.25%) | 0.238 | 3 (21.43%) | 3 (21.43%) | 1.000 |
|  | Automated peritoneal dialysis, n (%) | 4 (3.51%) | 2 (6.67%) | 2 (2.38%) | 0.752 | 1 (6.67%) | 1 (6.67%) | 1.000 |
| Laboratory variables | |  |  |  |  |  |  |  |
|  | Hemoglobin (g/L) | 103.75 ± 18.46 | 105.17 ± 18.59 | 103.27 ± 18.51 | 0.648 | 106.51 ± 21.03 | 104.80 ± 21.94 | 0.829 |
|  | Serum albumin (g/L) | 35.02 ± 5.59 | 34.04 ± 5.25 | 35.33 ± 5.69 | 0.300 | 35.03 ± 4.39 | 32.77 ± 6.01 | 0.250 |
|  | Albumin (<35g/L), n (%) | 45 (43.27%) | 11 (44.00%) | 34 (43.04%) | 1.000 | 6 (40.00%) | 9 (60.00%) | 0.465 |
|  | Triglyceride (mmol/L) | 2.13 ± 1.94 | 1.93 ± 1.98 | 2.19 ± 1.93 | 0.606 | 2.19 ± 2.37 | 1.55 ± 0.89 | 0.381 |
|  | Total cholesterol (mmol/L) | 4.65 ± 1.38 | 4.96 ± 1.08 | 4.55 ± 1.45 | 0.176 | 5.02 ± 0.99 | 4.02 ± 0.77 | 0.011^*^ |
|  | Serum calcium (mmol/L) | 2.20 ± 0.24 | 2.26 ± 0.17 | 2.19 ± 0.25 | 0.114 | 2.33 ± 0.13 | 2.20 ± 0.21 | 0.054 |
|  | Serum phosphorus (mmol/L) | 1.60 ± 0.50 | 1.53 ± 0.50 | 1.62 ± 0.50 | 0.492 | 1.50 ± 0.52 | 1.41 ± 0.42 | 0.635 |
|  | Serum potassium (mmol/L) | 4.09 ± 0.79 | 3.58 ± 0.79 | 4.24 ± 0.72 | 0.001^*^ | 3.82 ± 0.88 | 4.33 ± 0.93 | 0.147 |
|  | Serum sodium (mmol/L) | 138.30 ± 6.26 | 139.07 ± 4.08 | 138.07 ± 6.78 | 0.386 | 139.97 ± 2.49 | 139.05 ± 4.53 | 0.504 |
| Effluent WCC | |  |  |  |  |  |  |  |
|  | WCC on day 1 (10^^6^/L) | 3180.00 (916.25, 6482.00) | 2817.00 (1433.00, 9102.00) | 3200.00 (726.50, 5850.00) | 0.500 | 5542.00 (2430.00, 9431.00) | 3750.00 (2099.00, 7440.00) | 0.663 |
|  | WCC on day 3 (10^^6^/L) | 143.00 (50.00, 945.00) | 233.00 (95.00, 2240.00) | 139.50 (30.75, 681.00) | 0.068 | 139.00 (97.50, 2780.50) | 257.00 (119.50, 614.00) | 0.663 |
|  | WCC on day 3 (>300/mm^3^), n (%) | 38 (37.62%) | 11 (44.00%) | 27 (35.53%) | 0.603 | 7 (46.67%) | 7 (46.67%) | 1.000 |
| Treatment failure, n (%) | | 26 (22.81%) | 11 (36.67%) | 15 (17.86%) | 0.035^*^ | 5 (33.33%) | 2 (13.33) | 0.390 |
|  | Peritonitis-associated death | 5 (4.46%) | 3 (10.71%) | 2 (2.38%) | 0.099 | 1 (6.67%) | 0 (0.00%) | 1.000 |
|  | Peritonitis-associated haemodialysis transfer | 21 (18.75%) | 8 (28.57%) | 13 (15.48%) | 0.208 | 4 (26.67%) | 2 (13.33%) | 0.651 |
| Subsequent peritonitis within 6 months | | 12 (10.53%) | 3 (10.00%) | 9 (10.71%) | 0.913 | 1 (6.67%) | 2 (13.33%) | 0.582 |

Abbreviations: BMI, body mass index; PD, peritoneal dialysis; WCC, white cell counts.

^*^*P* < 0.05 or 0.001 between groups

**Supplement Table 4. Comparison of clinical characteristics and peritonitis related information between third-generation cephalosporins and other antibiotics for Gram-negative bacterial peritonitis within the vancomycin-based subgroup.**

|  | |  | Before matching | | | After matching | | |
| --- | --- | --- | --- | --- | --- | --- | --- | --- |
|  | | Total  (n=116) | Other antibiotics  (n=41) | Third-generation cephalosporin  (n=75) | *P*-value | Other antibiotics  (n=19) | Third-generation cephalosporin  (n=19) | *P*-value |
| Clinical characteristics | |  |  |  |  |  |  |  |
|  | Age (years) | 56.79 ± 14.15 | 51.45 ± 14.87 | 59.70 ± 12.94 | 0.004^*^ | 55.40 ± 16.93 | 57.53 ± 12.83 | 0.665 |
|  | Age (>60 years), n (%) | 56 (48.28%) | 12 (29.27%) | 44 (58.67%) | 0.005^*^ | 9 (47.37%) | 9 (47.37%) | 1.000 |
|  | Male, n (%) | 49 (42.24%) | 15 (36.59%) | 34 (45.33%) | 0.474 | 9 (47.37%) | 9 (47.37%) | 1.000 |
|  | BMI (kg/m2) | 23.08 ± 3.19 | 22.66 ± 3.67 | 23.31 ± 2.91 | 0.330 | 22.98 ± 3.51 | 23.14 ± 2.85 | 0.882 |
|  | PD duration (month) | 31.13 (16.17, 55.69) | 26.17 (7.60, 46.43) | 34.13 (17.95, 61.00) | 0.048^*^ | 26.17 (17.65, 33.05) | 26.57 (14.90, 49.74) | 0.930 |
|  | PD duration (>25 months), n (%) | 73 (62.93%) | 23 (56.10%) | 50 (66.67%) | 0.355 | 12 (63.16%) | 10 (52.63%) | 0.742 |
|  | Annual income, (>50,000¥), n (%) | 52 (45.61%) | 7 (17.07%) | 45 (61.64%) | <0.001^*^ | 5 (26.32%) | 5 (26.32%) | 1.000 |
|  | Cardiovascular disease, n (%) | 44 (37.93%) | 11 (26.83%) | 33 (44.00%) | 0.105 | 6 (31.58%) | 9 (47.37%) | 0.507 |
|  | Diabetes mellitus, n (%) | 50 (43.10%) | 13 (31.71%) | 37 (49.33%) | 0.102 | 11 (57.89%) | 11 (57.89%) | 1.000 |
|  | Peritonitis history, n (%) | 30 (25.86%) | 5 (12.20%) | 25 (33.33%) | 0.024^*^ | 3 (15.79%) | 2 (10.53%) | 1.000 |
|  | Automated peritoneal dialysis, n (%) | 3 (2.59%) | 2 (4.88%) | 1 (1.33%) | 0.670 | 1 (5.26%) | 1 (5.26%) | 1.000 |
| Laboratory variables | |  |  |  |  |  |  |  |
|  | Hemoglobin (g/L) | 108.93 ± 16.66 | 104.85 ± 17.46 | 111.14 ± 15.90 | 0.066 | 104.42 ± 17.57 | 115.06 ± 16.77 | 0.068 |
|  | Albumin (<35g/L), n (%) | 42 (37.50%) | 16 (41.03%) | 26 (35.62%) | 0.720 | 10 (52.63%) | 10 (52.63%) | 1.000 |
|  | Serum albumin (g/L) | 35.36 ± 5.16 | 35.28 ± 5.40 | 35.41 ± 5.06 | 0.903 | 34.45 ± 5.75 | 35.31 ± 6.61 | 0.673 |
|  | Triglyceride (mmol/L) | 2.16 ± 1.60 | 2.06 ± 1.59 | 2.21 ± 1.61 | 0.690 | 1.45 ± 0.59 | 1.72 ± 1.09 | 0.457 |
|  | Total cholesterol (mmol/L) | 4.77 ± 1.43 | 4.98 ± 1.52 | 4.65 ± 1.38 | 0.306 | 5.00 ± 1.66 | 4.66 ± 1.23 | 0.538 |
|  | Serum calcium (mmol/L) | 2.26 ± 0.21 | 2.22 ± 0.19 | 2.29 ± 0.21 | 0.108 | 2.26 ± 0.14 | 2.22 ± 0.22 | 0.535 |
|  | Serum phosphorus (mmol/L) | 1.51 ± 0.47 | 1.42 ± 0.42 | 1.56 ± 0.50 | 0.133 | 1.36 ± 0.42 | 1.57 ± 0.63 | 0.262 |
|  | Serum potassium (mmol/L) | 4.09 ± 0.69 | 4.17 ± 0.69 | 4.05 ± 0.69 | 0.388 | 4.32 ± 0.85 | 4.06 ± 0.95 | 0.405 |
|  | Serum sodium (mmol/L) | 138.08 ± 3.77 | 138.37 ± 3.19 | 137.93 ± 4.07 | 0.533 | 137.92 ± 3.08 | 138.69 ± 3.39 | 0.483 |
| Effluent WBC | |  |  |  |  |  |  |  |
|  | WBC on day 1 (10^^6^/L) | 2950.00 (1100.00, 6025.50) | 2340.00 (1235.00, 4780.00) | 3200.00 (856.00, 6205.00) | 0.579 | 3500.00 (2340.00, 6302.00) | 4470.00 (1600.00, 6332.50) | 0.965 |
|  | WBC on day 3 (10^^6^/L) | 752.00 (188.50, 3461.50) | 423.00 (97.00, 1853.25) | 1280.00 (216.00, 4152.00) | 0.045^*^ | 726.00 (211.00, 3560.00) | 1280.00 (264.50, 3230.50) | 0.804 |
|  | WBC on day 3 (>300/mm^3^), n (%) | 69 (62.16%) | 20 (52.63%) | 49 (67.12%) | 0.198 | 12 (63.16%) | 14 (73.68%) | 0.727 |
| Treatment failure, n (%) | | 30 (25.87%) | 8 (19.51%) | 22 (29.33%) | 0.248 | 4 (21.09%) | 5 (26.32%) | 1.000 |
|  | Peritonitis-associated death | 5 (4.39%) | 1 (2.44%) | 4 (5.48%) | 0.653 | 1 (5.26%) | 2 (10.53%) | 1.000 |
|  | Peritonitis-associated haemodialysis transfer | 25 (21.93%) | 7 (17.07%) | 18 (24.66%) | 0.482 | 3 (15.79%) | 3 (15.79%) | 1.000 |
| Subsequent peritonitis within 6 months | | 12 (10.34%) | 3 (7.32%) | 6 (8.00%) | 1.000 | 1 (5.26%) | 2 (10.53%) | 0.879 |

Abbreviations: BMI, body mass index; PD, peritoneal dialysis; WCC, white cell counts.

^*^*P* < 0.05 or 0.001 between groups; ^**^


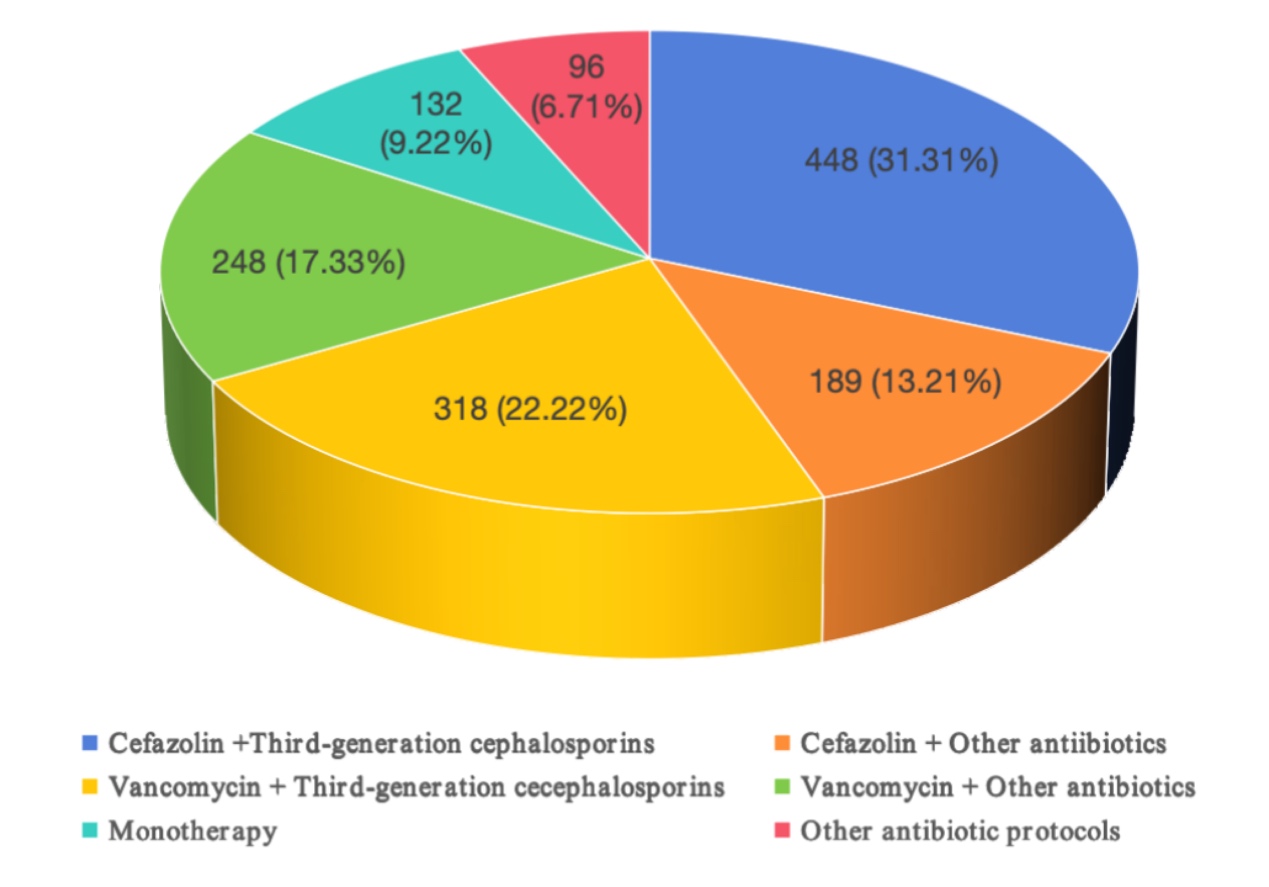


**Supplement Figure 1. The distribution of empirical antibiotic regimens in the cohort.**


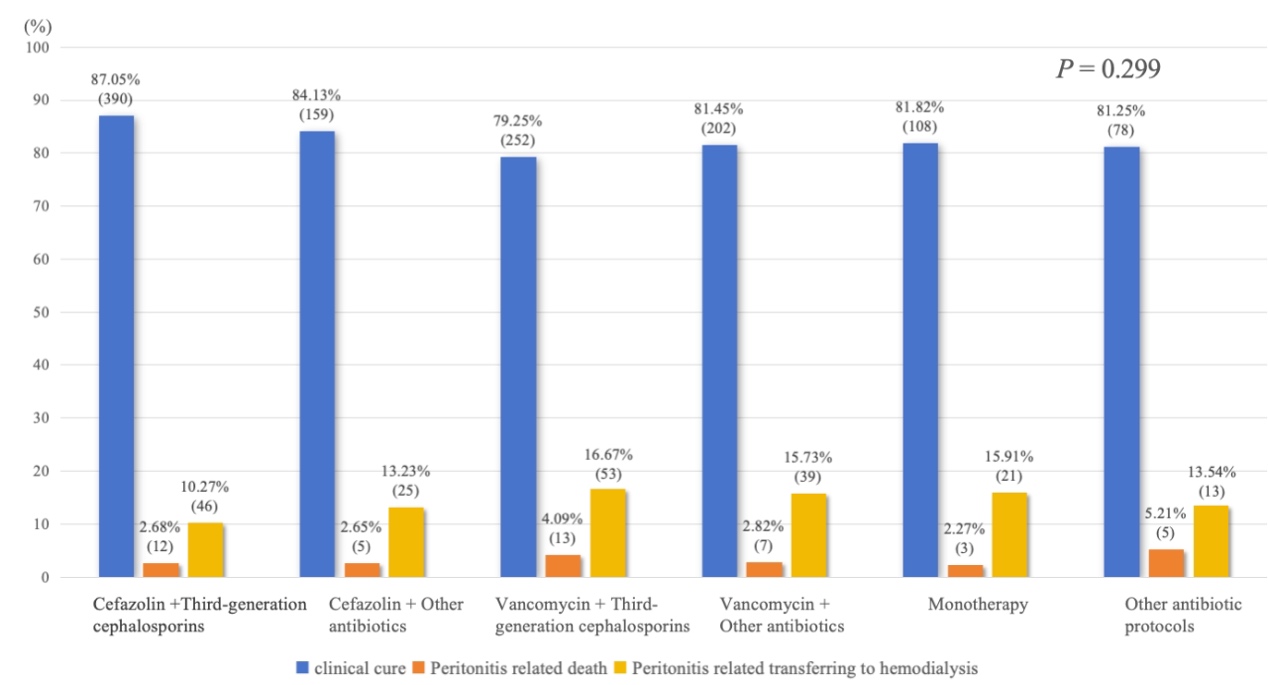


**Supplement Figure 2. The outcomes of peritonitis with different empirical antibiotic regimens.**
